# Clinical Studies of Disease in Children

**Published:** 1890-09

**Authors:** Eliza L. Walker Dunbar


### Clinical Records.

---

### CLINICAL STUDIES OF DISEASE IN CHILDREN.

By Eliza L. Walker Dunbar, M.D. *Zürich*, L.K.Q.C.P.I.

---

### PARTIAL BRONZE SKIN ASSOCIATED WITH INVOLUNTARY MUSCULAR ACTION AFTER A FALL ON THE LEFT SIDE.

Ada P., eight years old, was brought to me by her mother, in the summer of 1889, for medical advice concerning an affection described as "going down." The child during the past twelve months had been subject to being compelled to bend down sideways, sometimes to the left, sometimes to the right, less frequently backwards and also forwards. The movements occurred once, twice, three times, and never more than seven times in the day, at intervals of a few days, and once the interval had been as long as fourteen days. The movements appeared, on close questioning, to be those that would be effected sideways by action of the quadratus lumborum muscle of both sides; backwards by the two longissimi dorsi muscles, and forwards by the recti abdominales muscles. As it was distinctly stated that the head was stretched back and bent forwards with the backwards and forward movements, and sometimes even separately, the muscles which continue for the head the separate movements of the vertebral column were also implicated in the involuntary contractions. The movements were not sudden jerks, but gradual, the body swaying down by degrees and keeping bent for a little time, then relaxing and allowing of reposition after a very few minutes. The child seems always to have been assisted to the upright position after the attack. Once it was over she seemed to be about as well as usual and would resume her play or lessons. Mention must be made of the fact that the child had stated to her mother, for the first and only time, a week previously that she sometimes saw things double one above another. But this symptom never recurred after I saw her.

The skin of the dorsal surfaces of both hands was very brown, of a deeper brown than exposure to sun in an English city usually produces: but on my assuming that the hands were sunburnt, the mother immediately stated their colour had been the same during the past winter; neighbours had remarked how strangely brown the child was. The discolouration extended nearly half-way up the forearms on their dorsal surface, and ended with a line as if a sleeve had usually there encircled pretty closely the arm; but the child had not worn any such sleeve. There was also a patch of brown colouring on the right side of the neck below the ear. The whole face was brownish, but much lighter than arm and neck; the skin over the arm and under the right eye was rather scaly. The child did not feel ill, had a good appetite, and slept well. Urine contained no albumen and appeared to be normal.

The history of the case was thus given by the mother: In July, 1888, the child, while taking part in a school-treat, had fallen down sideways to the left over a stone. When picked up she continued bent to the left side and could not draw herself up straight. Carried to the accident ward of a local institution, she was literally put straight by the attendant surgeon and bandaged. There was no bruise to be seen either then or eight days afterwards, when the bandage was removed and the child dismissed as well. Two months afterwards, at the end of September, the involuntary muscular contractions began. The child was seen to bend down to the left side; the movement was repeated after a short time, and recurred again after a few days' interval. The brown colour (of neck, arms, hands, and face) was first noticed as being peculiar when it could no longer be ascribed to sunburn.

The previous history of the child showed that she had suffered from the age of 18 months to 3½ years from œdema, due probably to some kidney-disorder. She recovered on removal to the seaside, and became strong and well. Returning to Clifton after a year and a half, she seemed to flag, but was always well enough to attend school.

Medical treatment did not appear to influence the affection in any way during the few weeks I prescribed for it. I lost sight of the case till the end of December, when her mother, reappearing for medical advice for an infant, made the surprising statement that Ada was quite well, having lost for over six weeks the habit of "going down;" and some time after the last "going down" the hands, neck, and face had recovered the tint natural to the rest of the skin.

This I verified on seeing the child. She had in the meantime been without medical treatment.

The case I believe to be one of mechanical injury by a fall on the left side: (1) to the left supra-renal body, giving rise to a slight attack of Addison's disease; and (2) to the 12th left dorsal and the 1st left lumbar nerves, which supply the principal muscles involved in the contractions. The connection of these nerves within the grey substance of the spinal cord accounts for the irritation acting on the nerves of the corresponding muscles of the right side, as well as for implication of the nerves of the muscles moving the head, which moreover often moves co-ordinately with the whole movement of the spinal column. Nerve-connections within the spinal cord may also be invoked to account for the action of the muscles of the eye producing temporary double sight.

---

### ENLARGEMENT OF LIVER, PROBABLY FROM DEPOSIT OF FAT.

A little girl three years old showed such an increasing enlargement of the abdomen as to lead to medical advice being sought. The enlargement on examination I found to be entirely due to increase of size of the liver, which reached in its lowest boundary to a line drawn across the navel. Traces of rickets were present, but in a slight degree, and there was no deformity of the thorax such as would be followed by displacement of the liver. The little girl had during the short period of her existence shown always an extraordinarily large appetite, to appease which she had frequently been found eating paper in large quantities—newspaper and also cardboard. According to the mother's statement, the child ate habitually largely, was unrestricted as to quantity at meal-times, and required as much as her brother who was 14 years old. Often the child procured food between meals. Latterly many dainties refused by an invalid in the house had been eaten up by the child. She was well, except for a little diarrhœa, which soon ceased when appropriate remedies were used. Also she had been seen to pant a little after running upstairs, a symptom which soon disappeared after treatment had begun.

Every organ was sound with the exception of the liver. Though it was enlarged, it was quite painless however pressed. Temperature was normal and urine contained no albumen.

Considering the history of the case, the enormous appetite and recent means of extra indulgence, the painlessness, and the healthy state of heart, lungs, and kidneys, I diagnosed accumulation of fat in the liver. The treatment consisted chiefly in restricting the diet, and after six months' time the liver was found to be of normal proportions.

---

